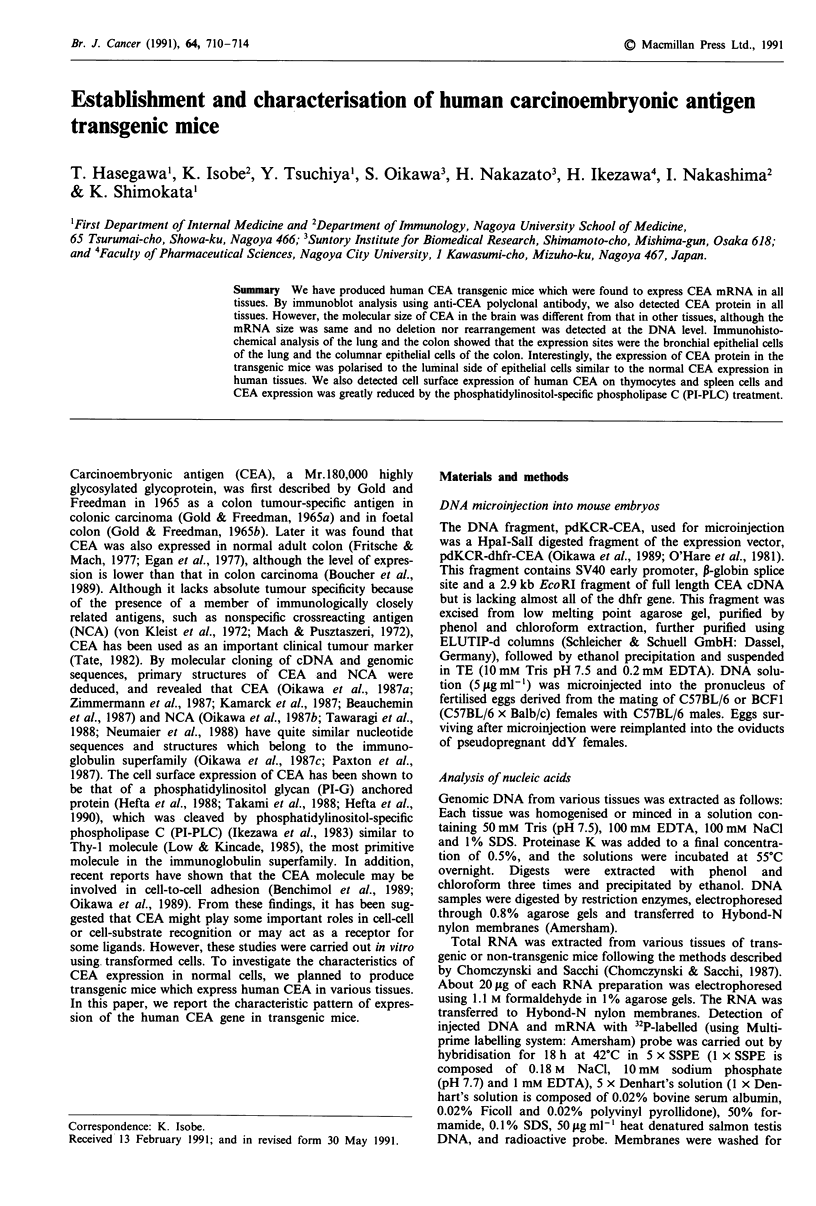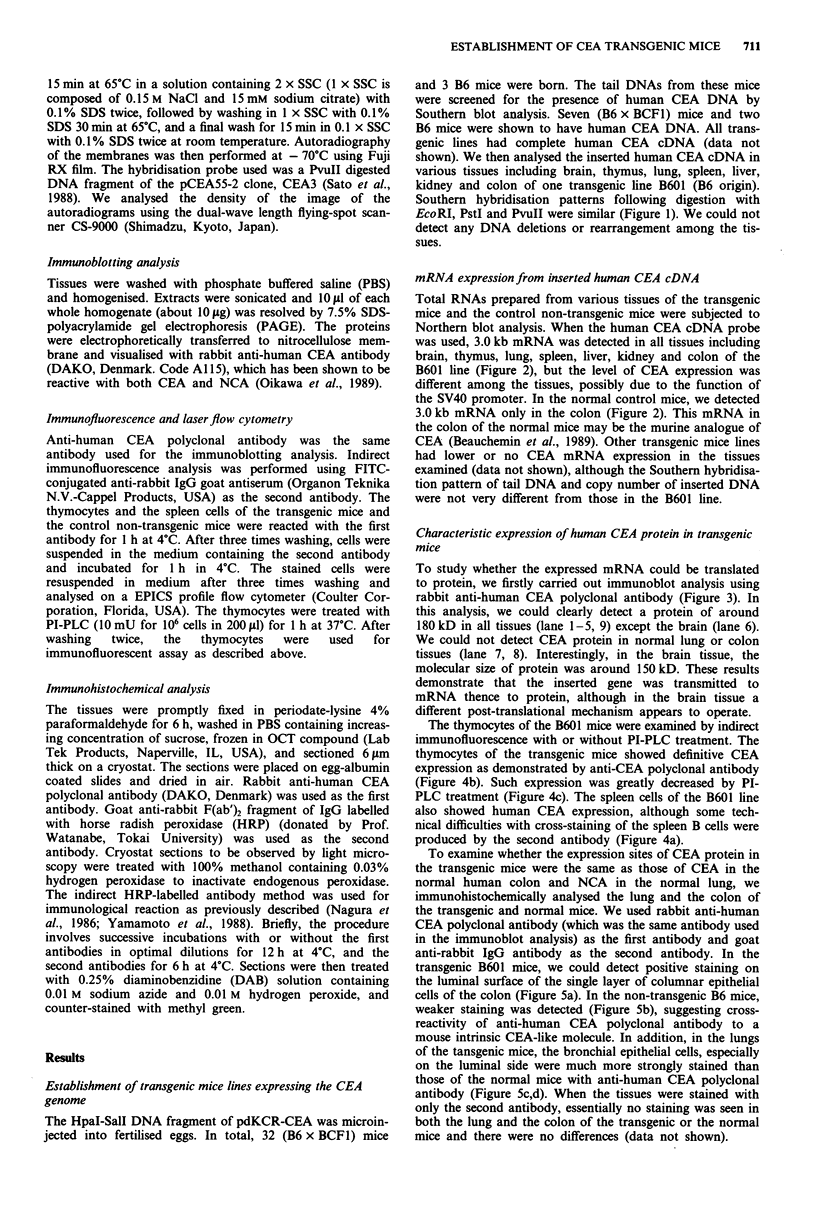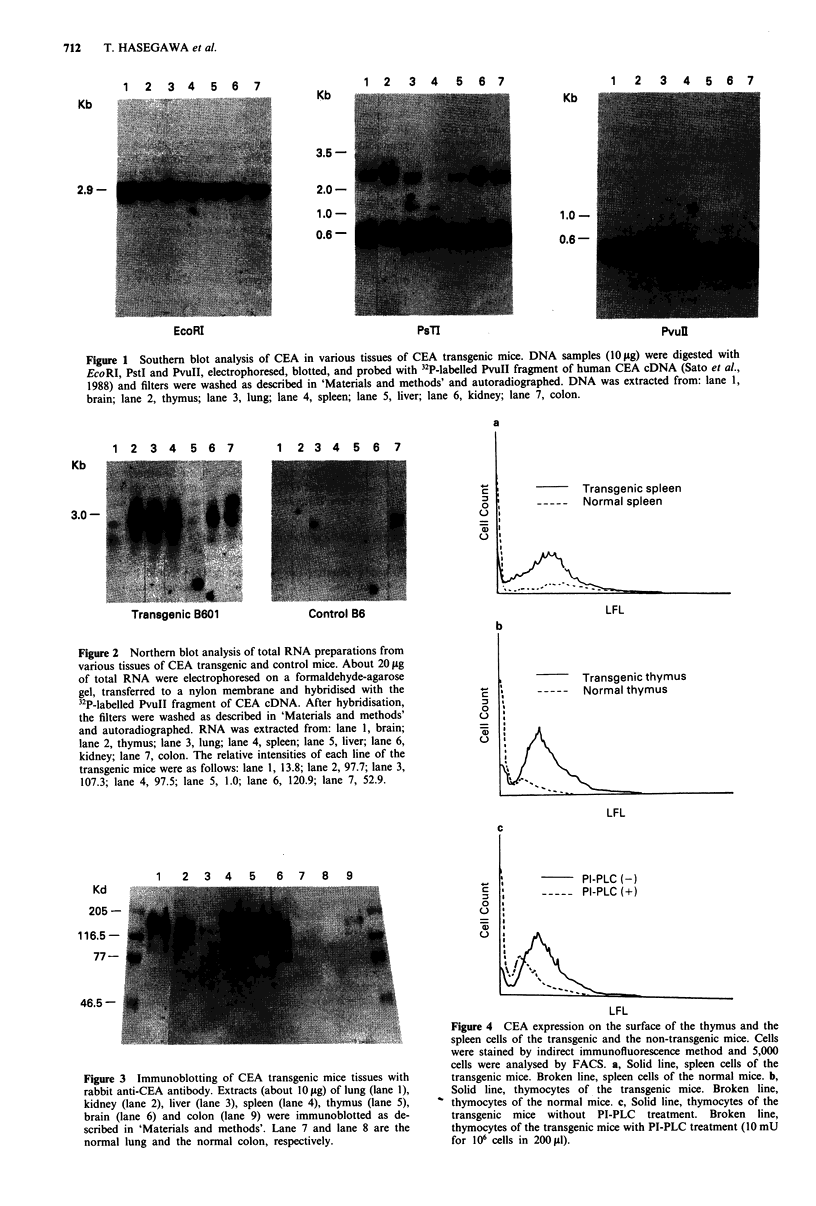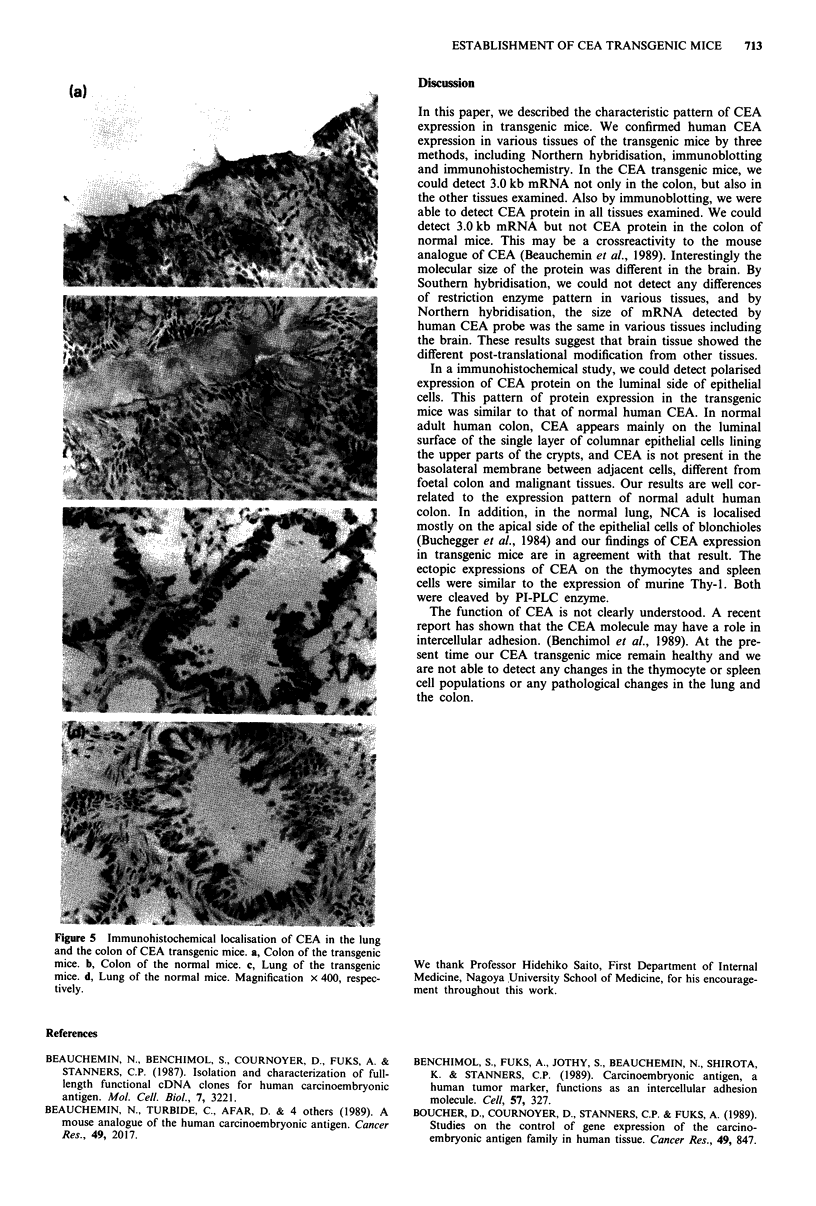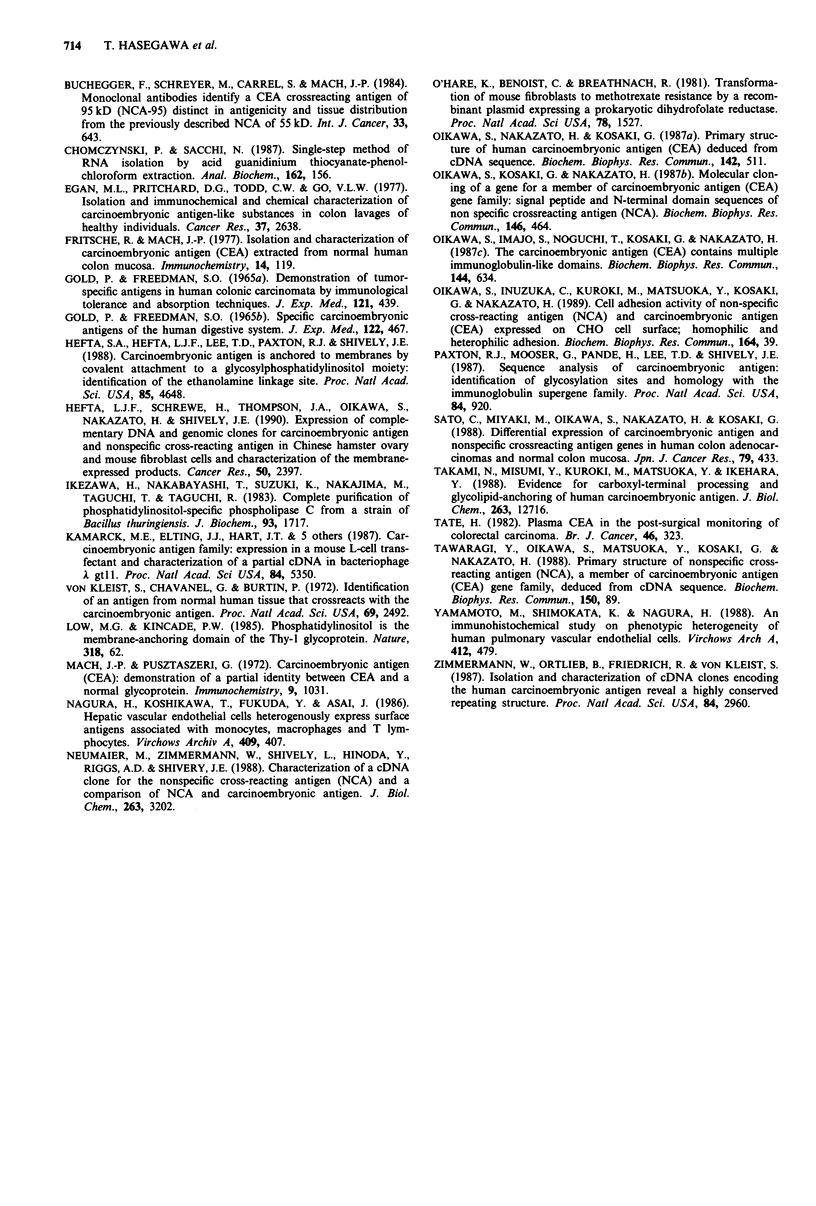# Establishment and characterisation of human carcinoembryonic antigen transgenic mice.

**DOI:** 10.1038/bjc.1991.386

**Published:** 1991-10

**Authors:** T. Hasegawa, K. Isobe, Y. Tsuchiya, S. Oikawa, H. Nakazato, H. Ikezawa, I. Nakashima, K. Shimokata

**Affiliations:** First Department of Internal Medicine, Nagoya University School of Medicine, Japan.

## Abstract

**Images:**


Br. J. Cancer (1991), 64, 710-714 © Macmillan Press Ltd., 1991

### Establishment and characterisation of human carcinoembryonic antigen transgenic mice

T. Hasegawa[1], K. Isobe[2], Y. Tsuchiya[1], S. Oikawa[3], H. Nakazato[3], H. Ikezawa[4], I. Nakashima[2] & K. Shimokata[1]

[1]*First Department of Internal Medicine and* [2]*Department of Immunology, Nagoya University School of Medicine, 65 Tsurumai-cho, Showa-ku, Nagoya 466;* [3]*Suntory Institute for Biomedical Research, Shimamoto-cho, Mishima-gun, Osaka 618; and* [4]*Faculty of Pharmaceutical Sciences, Nagoya City University, 1 Kawasumi-cho, Mizuho-ku, Nagoya 467, Japan.*

**Summary** We have produced human CEA transgenic mice which were found to express CEA mRNA in all tissues. By immunoblot analysis using anti-CEA polyclonal antibody, we also detected CEA protein in all tissues. However, the molecular size of CEA in the brain was different from that in other tissues, although the mRNA size was same and no deletion nor rearrangement was detected at the DNA level. Immunohistochemical analysis of the lung and the colon showed that the expression sites were the bronchial epithelial cells of the lung and the columnar epithelial cells of the colon. Interestingly, the expression of CEA protein in the transgenic mice was polarised to the luminal side of epithelial cells similar to the normal CEA expression in human tissues. We also detected cell surface expression of human CEA on thymocytes and spleen cells and CEA expression was greatly reduced by the phosphatidylinositol-specific phospholipase C (PI-PLC) treatment.

Carcinoembryonic antigen (CEA), a Mr.180,000 highly glycosylated glycoprotein, was first described by Gold and Freedman in 1965 as a colon tumour-specific antigen in colonic carcinoma (Gold & Freedman, 1965*a*) and in foetal colon (Gold & Freedman, 1965*b*). Later it was found that CEA was also expressed in normal adult colon (Fritsche & Mach, 1977; Egan *et al.*, 1977), although the level of expression is lower than that in colon carcinoma (Boucher *et al.*, 1989). Although it lacks absolute tumour specificity because of the presence of a member of immunologically closely related antigens, such as nonspecific crossreacting antigen (NCA) (von Kleist *et al.*, 1972; Mach & Pusztaszeri, 1972), CEA has been used as an important clinical tumour marker (Tate, 1982). By molecular cloning of cDNA and genomic sequences, primary structures of CEA and NCA were deduced, and revealed that CEA (Oikawa *et al.*, 1987*a*; Zimmermann *et al.*, 1987; Kamarck *et al.*, 1987; Beauchemin *et al.*, 1987) and NCA (Oikawa *et al.*, 1987*b*; Tawaragi *et al.*, 1988; Neumaier *et al.*, 1988) have quite similar nucleotide sequences and structures which belong to the immunoglobulin superfamily (Oikawa *et al.*, 1987*c*; Paxton *et al.*, 1987). The cell surface expression of CEA has been shown to be that of a phosphatidylinositol glycan (PI-G) anchored protein (Hefta *et al.*, 1988; Takami *et al.*, 1988; Hefta *et al.*, 1990), which was cleaved by phosphatidylinositol-specific phospholipase C (PI-PLC) (Ikezawa *et al.*, 1983) similar to Thy-1 molecule (Low & Kincade, 1985), the most primitive molecule in the immunoglobulin superfamily. In addition, recent reports have shown that the CEA molecule may be involved in cell-to-cell adhesion (Benchimol *et al.*, 1989; Oikawa *et al.*, 1989). From these findings, it has been suggested that CEA might play some important roles in cell-cell or cell-substrate recognition or may act as a receptor for some ligands. However, these studies were carried out *in vitro* using transformed cells. To investigate the characteristics of CEA expression in normal cells, we planned to produce transgenic mice which express human CEA in various tissues. In this paper, we report the characteristic pattern of expression of the human CEA gene in transgenic mice.

Correspondence: K. Isobe.
Received 13 February 1991; and in revised form 30 May 1991.

### Materials and methods

### *DNA microinjection into mouse embryos*

The DNA fragment, pdKCR-CEA, used for microinjection was a HpaI-SalI digested fragment of the expression vector, pdKCR-dhfr-CEA (Oikawa *et al.*, 1989; O'Hare *et al.*, 1981). This fragment contains SV40 early promoter, β-globin splice site and a 2.9 kb *Eco*RI fragment of full length CEA cDNA but is lacking almost all of the dhfr gene. This fragment was excised from low melting point agarose gel, purified by phenol and chloroform extraction, further purified using ELUTIP-d columns (Schleicher & Schuell GmbH: Dassel, Germany), followed by ethanol precipitation and suspended in TE (10 mM Tris pH 7.5 and 0.2 mM EDTA). DNA solution ($5 \mu g\,ml^{-1}$) was microinjected into the pronucleus of fertilised eggs derived from the mating of C57BL/6 or BCF1 (C57BL/6 × Balb/c) females with C57BL/6 males. Eggs surviving after microinjection were reimplanted into the oviducts of pseudopregnant ddY females.

### *Analysis of nucleic acids*

Genomic DNA from various tissues was extracted as follows: Each tissue was homogenised or minced in a solution containing 50 mM Tris (pH 7.5), 100 mM EDTA, 100 mM NaCl and 1% SDS. Proteinase K was added to a final concentration of 0.5%, and the solutions were incubated at 55°C overnight. Digests were extracted with phenol and chloroform three times and precipitated by ethanol. DNA samples were digested by restriction enzymes, electrophoresed through 0.8% agarose gels and transferred to Hybond-N nylon membranes (Amersham).

Total RNA was extracted from various tissues of transgenic or non-transgenic mice following the methods described by Chomczynski and Sacchi (Chomczynski & Sacchi, 1987). About 20 μg of each RNA preparation was electrophoresed using 1.1 M formaldehyde in 1% agarose gels. The RNA was transferred to Hybond-N nylon membranes. Detection of injected DNA and mRNA with $^{32}P$-labelled (using Multiprime labelling system: Amersham) probe was carried out by hybridisation for 18 h at 42°C in 5 × SSPE (1 × SSPE is composed of 0.18 M NaCl, 10 mM sodium phosphate (pH 7.7) and 1 mM EDTA), 5 × Denhart's solution (1 × Denhart's solution is composed of 0.02% bovine serum albumin, 0.02% Ficoll and 0.02% polyvinyl pyrollidone), 50% formamide, 0.1% SDS, $50 \mu g\,ml^{-1}$ heat denatured salmon testis DNA, and radioactive probe. Membranes were washed for

15 min at 65°C in a solution containing 2 × SSC (1 × SSC is composed of 0.15 M NaCl and 15 mM sodium citrate) with 0.1% SDS twice, followed by washing in 1 × SSC with 0.1% SDS 30 min at 65°C, and a final wash for 15 min in 0.1 × SSC with 0.1% SDS twice at room temperature. Autoradiography of the membranes was then performed at – 70°C using Fuji RX film. The hybridisation probe used was a PvuII digested DNA fragment of the pCEA55-2 clone, CEA3 (Sato *et al.*, 1988). We analysed the density of the image of the autoradiograms using the dual-wave length flying-spot scanner CS-9000 (Shimadzu, Kyoto, Japan).

*Immunoblotting analysis*

Tissues were washed with phosphate buffered saline (PBS) and homogenised. Extracts were sonicated and 10 µl of each whole homogenate (about 10 µg) was resolved by 7.5% SDS-polyacrylamide gel electrophoresis (PAGE). The proteins were electrophoretically transferred to nitrocellulose membrane and visualised with rabbit anti-human CEA antibody (DAKO, Denmark. Code A115), which has been shown to be reactive with both CEA and NCA (Oikawa *et al.*, 1989).

*Immunofluorescence and laser flow cytometry*

Anti-human CEA polyclonal antibody was the same antibody used for the immunoblotting analysis. Indirect immunofluorescence analysis was performed using FITC-conjugated anti-rabbit IgG goat antiserum (Organon Teknika N.V.-Cappel Products, USA) as the second antibody. The thymocytes and the spleen cells of the transgenic mice and the control non-transgenic mice were reacted with the first antibody for 1 h at 4°C. After three times washing, cells were suspended in the medium containing the second antibody and incubated for 1 h in 4°C. The stained cells were resuspended in medium after three times washing and analysed on a EPICS profile flow cytometer (Coulter Corporation, Florida, USA). The thymocytes were treated with PI-PLC (10 mU for $10^6$ cells in 200 µl) for 1 h at 37°C. After washing twice, the thymocytes were used for immunofluorescent assay as described above.

*Immunohistochemical analysis*

The tissues were promptly fixed in periodate-lysine 4% paraformaldehyde for 6 h, washed in PBS containing increasing concentration of sucrose, frozen in OCT compound (Lab Tek Products, Naperville, IL, USA), and sectioned 6 µm thick on a cryostat. The sections were placed on egg-albumin coated slides and dried in air. Rabbit anti-human CEA polyclonal antibody (DAKO, Denmark) was used as the first antibody. Goat anti-rabbit $F(ab')_2$ fragment of IgG labelled with horse radish peroxidase (HRP) (donated by Prof. Watanabe, Tokai University) was used as the second antibody. Cryostat sections to be observed by light microscopy were treated with 100% methanol containing 0.03% hydrogen peroxidase to inactivate endogenous peroxidase. The indirect HRP-labelled antibody method was used for immunological reaction as previously described (Nagura *et al.*, 1986; Yamamoto *et al.*, 1988). Briefly, the procedure involves successive incubations with or without the first antibodies in optimal dilutions for 12 h at 4°C, and the second antibodies for 6 h at 4°C. Sections were then treated with 0.25% diaminobenzidine (DAB) solution containing 0.01 M sodium azide and 0.01 M hydrogen peroxide, and counter-stained with methyl green.

### Results

*Establishment of transgenic mice lines expressing the CEA genome*

The HpaI-SalI DNA fragment of pdKCR-CEA was microinjected into fertilised eggs. In total, 32 (B6 × BCF1) mice and 3 B6 mice were born. The tail DNAs from these mice were screened for the presence of human CEA DNA by Southern blot analysis. Seven (B6 × BCF1) mice and two B6 mice were shown to have human CEA DNA. All transgenic lines had complete human CEA cDNA (data not shown). We then analysed the inserted human CEA cDNA in various tissues including brain, thymus, lung, spleen, liver, kidney and colon of one transgenic line B601 (B6 origin). Southern hybridisation patterns following digestion with *Eco*RI, PstI and PvuII were similar (Figure 1). We could not detect any DNA deletions or rearrangement among the tissues.

*mRNA expression from inserted human CEA cDNA*

Total RNAs prepared from various tissues of the transgenic mice and the control non-transgenic mice were subjected to Northern blot analysis. When the human CEA cDNA probe was used, 3.0 kb mRNA was detected in all tissues including brain, thymus, lung, spleen, liver, kidney and colon of the B601 line (Figure 2), but the level of CEA expression was different among the tissues, possibly due to the function of the SV40 promoter. In the normal control mice, we detected 3.0 kb mRNA only in the colon (Figure 2). This mRNA in the colon of the normal mice may be the murine analogue of CEA (Beauchemin *et al.*, 1989). Other transgenic mice lines had lower or no CEA mRNA expression in the tissues examined (data not shown), although the Southern hybridisation pattern of tail DNA and copy number of inserted DNA were not very different from those in the B601 line.

*Characteristic expression of human CEA protein in transgenic mice*

To study whether the expressed mRNA could be translated to protein, we firstly carried out immunoblot analysis using rabbit anti-human CEA polyclonal antibody (Figure 3). In this analysis, we could clearly detect a protein of around 180 kD in all tissues (lane 1–5, 9) except the brain (lane 6). We could not detect CEA protein in normal lung or colon tissues (lane 7, 8). Interestingly, in the brain tissue, the molecular size of protein was around 150 kD. These results demonstrate that the inserted gene was transmitted to mRNA thence to protein, although in the brain tissue a different post-translational mechanism appears to operate.

The thymocytes of the B601 mice were examined by indirect immunofluorescence with or without PI-PLC treatment. The thymocytes of the transgenic mice showed definitive CEA expression as demonstrated by anti-CEA polyclonal antibody (Figure 4b). Such expression was greatly decreased by PI-PLC treatment (Figure 4c). The spleen cells of the B601 line also showed human CEA expression, although some technical difficulties with cross-staining of the spleen B cells were produced by the second antibody (Figure 4a).

To examine whether the expression sites of CEA protein in the transgenic mice were the same as those of CEA in the normal human colon and NCA in the normal lung, we immunohistochemically analysed the lung and the colon of the transgenic and normal mice. We used rabbit anti-human CEA polyclonal antibody (which was the same antibody used in the immunoblot analysis) as the first antibody and goat anti-rabbit IgG antibody as the second antibody. In the transgenic B601 mice, we could detect positive staining on the luminal surface of the single layer of columnar epithelial cells of the colon (Figure 5a). In the non-transgenic B6 mice, weaker staining was detected (Figure 5b), suggesting cross-reactivity of anti-human CEA polyclonal antibody to a mouse intrinsic CEA-like molecule. In addition, in the lungs of the tansgenic mice, the bronchial epithelial cells, especially on the luminal side were much more strongly stained than those of the normal mice with anti-human CEA polyclonal antibody (Figure 5c,d). When the tissues were stained with only the second antibody, essentially no staining was seen in both the lung and the colon of the transgenic or the normal mice and there were no differences (data not shown).

**Figure 1** Southern blot analysis of CEA in various tissues of CEA transgenic mice. DNA samples (10 µg) were digested with *Eco*RI, PstI and PvuII, electrophoresed, blotted, and probed with $^{32}$P-labelled PvuII fragment of human CEA cDNA (Sato *et al.*, 1988) and filters were washed as described in 'Materials and methods' and autoradiographed. DNA was extracted from: lane 1, brain; lane 2, thymus; lane 3, lung; lane 4, spleen; lane 5, liver; lane 6, kidney; lane 7, colon.

**Figure 2** Northern blot analysis of total RNA preparations from various tissues of CEA transgenic and control mice. About 20 µg of total RNA were electrophoresed on a formaldehyde-agarose gel, transferred to a nylon membrane and hybridised with the $^{32}$P-labelled PvuII fragment of CEA cDNA. After hybridisation, the filters were washed as described in 'Materials and methods' and autoradiographed. RNA was extracted from: lane 1, brain; lane 2, thymus; lane 3, lung; lane 4, spleen; lane 5, liver; lane 6, kidney; lane 7, colon. The relative intensities of each line of the transgenic mice were as follows: lane 1, 13.8; lane 2, 97.7; lane 3, 107.3; lane 4, 97.5; lane 5, 1.0; lane 6, 120.9; lane 7, 52.9.

**Figure 3** Immunoblotting of CEA transgenic mice tissues with rabbit anti-CEA antibody. Extracts (about 10 µg) of lung (lane 1), kidney (lane 2), liver (lane 3), spleen (lane 4), thymus (lane 5), brain (lane 6) and colon (lane 9) were immunoblotted as described in 'Materials and methods'. Lane 7 and lane 8 are the normal lung and the normal colon, respectively.

**Figure 4** CEA expression on the surface of the thymus and the spleen cells of the transgenic and the non-transgenic mice. Cells were stained by indirect immunofluorescence method and 5,000 cells were analysed by FACS. **a**, Solid line, spleen cells of the transgenic mice. Broken line, spleen cells of the normal mice. **b**, Solid line, thymocytes of the transgenic mice. Broken line, thymocytes of the normal mice. **c**, Solid line, thymocytes of the transgenic mice without PI-PLC treatment. Broken line, thymocytes of the transgenic mice with PI-PLC treatment (10 mU for $10^6$ cells in 200 µl).

**Figure 5** Immunohistochemical localisation of CEA in the lung and the colon of CEA transgenic mice. **a**, Colon of the transgenic mice. **b**, Colon of the normal mice. **c**, Lung of the transgenic mice. **d**, Lung of the normal mice. Magnification × 400, respectively.

### Discussion

In this paper, we described the characteristic pattern of CEA expression in transgenic mice. We confirmed human CEA expression in various tissues of the transgenic mice by three methods, including Northern hybridisation, immunoblotting and immunohistochemistry. In the CEA transgenic mice, we could detect 3.0 kb mRNA not only in the colon, but also in the other tissues examined. Also by immunoblotting, we were able to detect CEA protein in all tissues examined. We could detect 3.0 kb mRNA but not CEA protein in the colon of normal mice. This may be a crossreactivity to the mouse analogue of CEA (Beauchemin *et al.*, 1989). Interestingly the molecular size of the protein was different in the brain. By Southern hybridisation, we could not detect any differences of restriction enzyme pattern in various tissues, and by Northern hybridisation, the size of mRNA detected by human CEA probe was the same in various tissues including the brain. These results suggest that brain tissue showed the different post-translational modification from other tissues.

In a immunohistochemical study, we could detect polarised expression of CEA protein on the luminal side of epithelial cells. This pattern of protein expression in the transgenic mice was similar to that of normal human CEA. In normal adult human colon, CEA appears mainly on the luminal surface of the single layer of columnar epithelial cells lining the upper parts of the crypts, and CEA is not present in the basolateral membrane between adjacent cells, different from foetal colon and malignant tissues. Our results are well correlated to the expression pattern of normal adult human colon. In addition, in the normal lung, NCA is localised mostly on the apical side of the epithelial cells of blonchioles (Buchegger *et al.*, 1984) and our findings of CEA expression in transgenic mice are in agreement with that result. The ectopic expressions of CEA on the thymocytes and spleen cells were similar to the expression of murine Thy-1. Both were cleaved by PI-PLC enzyme.

The function of CEA is not clearly understood. A recent report has shown that the CEA molecule may have a role in intercellular adhesion. (Benchimol *et al.*, 1989). At the present time our CEA transgenic mice remain healthy and we are not able to detect any changes in the thymocyte or spleen cell populations or any pathological changes in the lung and the colon.

We thank Professor Hidehiko Saito, First Department of Internal Medicine, Nagoya University School of Medicine, for his encouragement throughout this work.

### References

BEAUCHEMIN, N., BENCHIMOL, S., COURNOYER, D., FUKS, A. & STANNERS, C.P. (1987). Isolation and characterization of full-length functional cDNA clones for human carcinoembryonic antigen. *Mol. Cell. Biol.*, **7**, 3221.

BEAUCHEMIN, N., TURBIDE, C., AFAR, D. & 4 others (1989). A mouse analogue of the human carcinoembryonic antigen. *Cancer Res.*, **49**, 2017.

BENCHIMOL, S., FUKS, A., JOTHY, S., BEAUCHEMIN, N., SHIROTA, K. & STANNERS, C.P. (1989). Carcinoembryonic antigen, a human tumor marker, functions as an intercellular adhesion molecule. *Cell*, **57**, 327.

BOUCHER, D., COURNOYER, D., STANNERS, C.P. & FUKS, A. (1989). Studies on the control of gene expression of the carcinoembryonic antigen family in human tissue. *Cancer Res.*, **49**, 847.

BUCHEGGER, F., SCHREYER, M., CARREL, S. & MACH, J.-P. (1984). Monoclonal antibodies identify a CEA crossreacting antigen of 95 kD (NCA-95) distinct in antigenicity and tissue distribution from the previously described NCA of 55 kD. *Int. J. Cancer*, **33**, 643.

CHOMCZYNSKI, P. & SACCHI, N. (1987). Single-step method of RNA isolation by acid guanidinium thiocyanate-phenol-chloroform extraction. *Anal. Biochem.*, **162**, 156.

EGAN, M.L., PRITCHARD, D.G., TODD, C.W. & GO, V.L.W. (1977). Isolation and immunochemical and chemical characterization of carcinoembryonic antigen-like substances in colon lavages of healthy individuals. *Cancer Res.*, **37**, 2638.

FRITSCHE, R. & MACH, J.-P. (1977). Isolation and characterization of carcinoembryonic antigen (CEA) extracted from normal human colon mucosa. *Immunochemistry*, **14**, 119.

GOLD, P. & FREEDMAN, S.O. (1965*a*). Demonstration of tumor-specific antigens in human colonic carcinomata by immunological tolerance and absorption techniques. *J. Exp. Med.*, **121**, 439.

GOLD, P. & FREEDMAN, S.O. (1965*b*). Specific carcinoembryonic antigens of the human digestive system. *J. Exp. Med.*, **122**, 467.

HEFTA, S.A., HEFTA, L.J.F., LEE, T.D., PAXTON, R.J. & SHIVELY, J.E. (1988). Carcinoembryonic antigen is anchored to membranes by covalent attachment to a glycosylphosphatidylinositol moiety: identification of the ethanolamine linkage site. *Proc. Natl Acad. Sci. USA*, **85**, 4648.

HEFTA, L.J.F., SCHREWE, H., THOMPSON, J.A., OIKAWA, S., NAKAZATO, H. & SHIVELY, J.E. (1990). Expression of complementary DNA and genomic clones for carcinoembryonic antigen and nonspecific cross-reacting antigen in Chinese hamster ovary and mouse fibroblast cells and characterization of the membrane-expressed products. *Cancer Res.*, **50**, 2397.

IKEZAWA, H., NAKABAYASHI, T., SUZUKI, K., NAKAJIMA, M., TAGUCHI, T. & TAGUCHI, R. (1983). Complete purification of phosphatidylinositol-specific phospholipase C from a strain of *Bacillus thuringiensis*. *J. Biochem.*, **93**, 1717.

KAMARCK, M.E., ELTING, J.J., HART, J.T. & 5 others (1987). Carcinoembryonic antigen family: expression in a mouse L-cell transfectant and characterization of a partial cDNA in bacteriophage λ gt11. *Proc. Natl Acad. Sci USA*, **84**, 5350.

VON KLEIST, S., CHAVANEL, G. & BURTIN, P. (1972). Identification of an antigen from normal human tissue that crossreacts with the carcinoembryonic antigen. *Proc. Natl Acad. Sci. USA*, **69**, 2492.

LOW, M.G. & KINCADE, P.W. (1985). Phosphatidylinositol is the membrane-anchoring domain of the Thy-1 glycoprotein. *Nature*, **318**, 62.

MACH, J.-P. & PUSZTASZERI, G. (1972). Carcinoembryonic antigen (CEA): demonstration of a partial identity between CEA and a normal glycoprotein. *Immunochemistry*, **9**, 1031.

NAGURA, H., KOSHIKAWA, T., FUKUDA, Y. & ASAI, J. (1986). Hepatic vascular endothelial cells heterogenously express surface antigens associated with monocytes, macrophages and T lymphocytes. *Virchows Archiv A*, **409**, 407.

NEUMAIER, M., ZIMMERMANN, W., SHIVELY, L., HINODA, Y., RIGGS, A.D. & SHIVERY, J.E. (1988). Characterization of a cDNA clone for the nonspecific cross-reacting antigen (NCA) and a comparison of NCA and carcinoembryonic antigen. *J. Biol. Chem.*, **263**, 3202.

O'HARE, K., BENOIST, C. & BREATHNACH, R. (1981). Transformation of mouse fibroblasts to methotrexate resistance by a recombinant plasmid expressing a prokaryotic dihydrofolate reductase. *Proc. Natl Acad. Sci USA*, **78**, 1527.

OIKAWA, S., NAKAZATO, H. & KOSAKI, G. (1987*a*). Primary structure of human carcinoembryonic antigen (CEA) deduced from cDNA sequence. *Biochem. Biophys. Res. Commun.*, **142**, 511.

OIKAWA, S., KOSAKI, G. & NAKAZATO, H. (1987*b*). Molecular cloning of a gene for a member of carcinoembryonic antigen (CEA) gene family: signal peptide and N-terminal domain sequences of non specific crossreacting antigen (NCA). *Biochem. Biophys. Res. Commun.*, **146**, 464.

OIKAWA, S., IMAJO, S., NOGUCHI, T., KOSAKI, G. & NAKAZATO, H. (1987*c*). The carcinoembryonic antigen (CEA) contains multiple immunoglobulin-like domains. *Biochem. Biophys. Res. Commun.*, **144**, 634.

OIKAWA, S., INUZUKA, C., KUROKI, M., MATSUOKA, Y., KOSAKI, G. & NAKAZATO, H. (1989). Cell adhesion activity of non-specific cross-reacting antigen (NCA) and carcinoembryonic antigen (CEA) expressed on CHO cell surface; homophilic and heterophilic adhesion. *Biochem. Biophys. Res. Commun.*, **164**, 39.

PAXTON, R.J., MOOSER, G., PANDE, H., LEE, T.D. & SHIVELY, J.E. (1987). Sequence analysis of carcinoembryonic antigen: identification of glycosylation sites and homology with the immunoglobulin supergene family. *Proc. Natl Acad. Sci. USA*, **84**, 920.

SATO, C., MIYAKI, M., OIKAWA, S., NAKAZATO, H. & KOSAKI, G. (1988). Differential expression of carcinoembryonic antigen and nonspecific crossreacting antigen genes in human colon adenocarcinomas and normal colon mucosa. *Jpn. J. Cancer Res.*, **79**, 433.

TAKAMI, N., MISUMI, Y., KUROKI, M., MATSUOKA, Y. & IKEHARA, Y. (1988). Evidence for carboxyl-terminal processing and glycolipid-anchoring of human carcinoembryonic antigen. *J. Biol. Chem.*, **263**, 12716.

TATE, H. (1982). Plasma CEA in the post-surgical monitoring of colorectal carcinoma. *Br. J. Cancer*, **46**, 323.

TAWARAGI, Y., OIKAWA, S., MATSUOKA, Y., KOSAKI, G. & NAKAZATO, H. (1988). Primary structure of nonspecific crossreacting antigen (NCA), a member of carcinoembryonic antigen (CEA) gene family, deduced from cDNA sequence. *Biochem. Biophys. Res. Commun.*, **150**, 89.

YAMAMOTO, M., SHIMOKATA, K. & NAGURA, H. (1988). An immunohistochemical study on phenotypic heterogeneity of human pulmonary vascular endothelial cells. *Virchows Arch A*, **412**, 479.

ZIMMERMANN, W., ORTLIEB, B., FRIEDRICH, R. & VON KLEIST, S. (1987). Isolation and characterization of cDNA clones encoding the human carcinoembryonic antigen reveal a highly conserved repeating structure. *Proc. Natl Acad. Sci. USA*, **84**, 2960.